# Bird species richness is associated with phylogenetic relatedness, plant species richness, and altitudinal range in Inner Mongolia

**DOI:** 10.1002/ece3.3606

**Published:** 2017-11-23

**Authors:** Chenxia Liang, Gang Feng, Xingfeng Si, Lingfeng Mao, Guisheng Yang, Jens‐Christian Svenning, Jie Yang

**Affiliations:** ^1^ School of Ecology and Environment Inner Mongolia University Hohhot China; ^2^ Department of Biological Sciences University of Toronto‐Scarborough Toronto ON Canada; ^3^ College of Life Sciences Zhejiang University Hangzhou China; ^4^ Department of Renewable Resources University of Alberta Edmonton AB Canada; ^5^ School of Life Sciences Inner Mongolia University Hohhot China; ^6^ Section for Ecoinformatics and Biodiversity Department of Bioscience Aarhus University Aarhus C Denmark

**Keywords:** biotic interaction, bird species richness, contemporary climate, environmental heterogeneity, glacial–interglacial climate change

## Abstract

Bird species richness is mediated by local, regional, and historical factors, for example, competition, environmental heterogeneity, contemporary, and historical climate. Here, we related bird species richness with phylogenetic relatedness of bird assemblages, plant species richness, topography, contemporary climate, and glacial‐interglacial climate change to investigate the relative importance of these factors. This study was conducted in Inner Mongolia, an arid and semiarid region with diverse vegetation types and strong species richness gradients. The following associated variables were included as follows: phylogenetic relatedness of bird assemblages (Net Relatedness Index, NRI), plant species richness, altitudinal range, contemporary climate (mean annual temperature and precipitation, MAT and MAP), and contemporary‐Last Glacial Maximum (LGM) change in climate (change in MAT and change in MAP). Ordinary least squares linear, simultaneous autoregressive linear, and Random Forest models were used to assess the associations between these variables and bird species richness across this region. We found that bird species richness was correlated negatively with NRI and positively with plant species richness and altitudinal range, with no significant correlations with contemporary climate and glacial–interglacial climate change. The six best combinations of variables ranked by Random Forest models consistently included NRI, plant species richness, and contemporary‐LGM change in MAT. Our results suggest important roles of local ecological factors in shaping the distribution of bird species richness across this semiarid region. Our findings highlight the potential importance of these local ecological factors, for example, environmental heterogeneity, habitat filtering, and biotic interactions, in biodiversity maintenance.

## 1 INTRODUCTION

Geographic distribution of species diversity and its drivers at broad scales is an important topic in ecology and biogeography (Brown, 2014; Currie, 1991; Fine, 2015). The underlying factors associated with species distribution include local (e.g., biotic interactions and habitat heterogeneity), regional (e.g., energy availability and water–energy dynamics), and historical (e.g., geological events and glacial–interglacial climate change) variables (Fine, 2015; Qu et al., 2015; Svenning, Eiserhardt, Normand, Ordonez, & Sandel, 2015).

Local ecological factors, including both biotic and abiotic factors, not only strongly constrain local community composition and structure, but could also affect species distribution at regional and global scales (Feng et al., 2016; Fine, 2015; Schemske, Mittelbach, Cornell, Sobel, & Roy, 2009). For instance, environmental heterogeneity, especially elevation range, is associated with patterns of species richness (Jetz & Rahbek, 2002; Kerr & Packer, 1997; Novillo & Ojeda, 2014). Availability of food resources and vegetation structure is also strongly associated with bird species richness at regional scales (Ferger, Schleuning, Hemp, Howell, & Böhning‐Gaese, 2014; Zhang, Kissling, & He, 2013). Both competition and facilitation among bird species could improve the prediction of bird species distribution at macro‐ecological scales (Heikkinen, Luoto, Virkkala, Pearson, & Körber, 2007; Laube, Graham, & Böhning‐Gaese, 2013; Pigot & Tobias, 2013).

In addition to those local ecological drivers, it has also been reported that regional and historical factors play important roles in shaping the geographic distribution of species richness at macro‐ecological scales, supporting the energy availability hypothesis, the water–energy dynamics hypothesis, the historical hypothesis, the refuge hypothesis, etc. (Davies et al., 2007; Fjeldså & Lovett, 1997; Li et al., 2013; Qu et al., 2015; Rahbek & Graves, 2001). Specifically, bird species richness is strongly associated with contemporary climate variables, such as precipitation, temperature, and actual evapotranspiration (Davies et al., 2007; Li et al., 2013; Rahbek & Graves, 2001). Glacial–interglacial refuge and geological events may also affect the distribution of species richness through their impact on species speciation and extinction (Fjeldså & Lovett, 1997; Qu et al., 2015).

With an area of 120 million ha (3.3 times the size of Germany and running 3,000 km from northwest to southeast), Inner Mongolia has a wide range of climate (e.g., mean annual temperature ranging from −2 to 6°C and mean annual precipitation ranging from 40 to 450 mm, Wu, Zhang, Li, & Liang, 2015). As a result, vegetation types, plant, and bird species are very diverse in Inner Mongolia, for example, there are forest, grassland, and desert, which are home to 2,447 known vascular plant species and 467 known bird species (Xu, 2007, 2015; Zhao, 2012), which provides an ideal system to investigate the geographic distribution of bird diversity. In this study, we assessed the associations between bird species richness and phylogenetic relatedness of bird assemblages, plant species richness, topography, contemporary climate, as well as glacial–interglacial climate change in Inner Mongolia (97°12′–126°04′E, 37°24′–53°23′N).

## 2 MATERIALS AND METHODS

### 2.1 Geographic data

Bird distribution data at the county scale were compiled from the third and fourth volumes of Fauna of Inner Mongolia (Xu, 2007, 2015). Plant distribution data at the county scale were collected from Chinese Vascular Plant Distribution Database, which was compiled from plant occurrence records in counties from *Flora Reipublicae Popularis Sinicae* (Delecti Florae Reipublicae Popularis Sinicae Agendae Academiae Sinicae, 1959), provincial and regional floras, as well as herbarium specimens. Plant species richness is interpreted as food resources and habitat diversity (Zhang et al., 2013). Eighty‐six counties were included (see Table S1 for more information), with areas ranging from 100 km^2^ to 90,000 km^2^ (area was not a factor significantly affecting bird species richness, Table 1).

**Table 1:** Results of single‐variable analysis by ordinary least squares (OLS) and simultaneous autoregressive (SAR) models. MAT and MAP are mean annual temperature and precipitation. Change_MAT_ and Change_MAP_ are the contemporary‐Last Glacial Maximum change in MAT and MAP. SR_plant_ is species richness of plants. ALT_range_ is altitudinal range. NRI is phylogenetic relatedness of bird assemblages. Coefficients (coef) and adjusted *r* ^2^ were given. All statistically significant *p*‐values were less than .01 and are indicated as *

	Coef_OLS_	*r* ^2^_OLS_	Coef_SAR_	*r* ^2^_SAR_
Area	0.06	−.01	0.09	.08
MAT	−0.05	−.01	0.02	.08
MAP	0.08	−.01	0.07	.08
Change_MAT_	−0.18	.02	−0.22	.10
Change_MAP_	0.20	.03	0.17	.09
SR_plant_	0.52	.26*	0.49	.30*
ALT_range_	0.37	.13*	0.34	.17*
NRI	−0.68	.45*	−0.67	.48*

### 2.2 Environmental data

Climate variables, mean annual temperature (MAT), mean annual precipitation (MAP), temperature in Last Glacial Maximum (MAT in LGM), precipitation in Last Glacial Maximum (MAP in LGM), and altitudinal range were collected from WorldClim (Hijmans, Cameron, Parra, Jones, & Jarvis, 2005). Altitudinal range is a proxy of environmental heterogeneity (Stein, Gerstner, & Kreft, 2014). MAT in LGM and MAP in LGM were extracted from the Community Climate System Model version 3 (CCSM3; Hijmans et al., 2005; Otto‐Bliesner et al., 2006) and the Model for Interdisciplinary Research on Climate version 3.2 (MIROC3.2; Hasumi & Emori, 2004). MAT in LGM and MAP in LGM were then summarized as the mean values of the two models. Change in MAT and change in MAP were calculated as contemporary values minus LGM values (Sandel et al., 2011).

### 2.3 Phylogeny

A distribution of 10,000 phylogenies was downloaded from the global phylogeny of birds (Jetz et al., 2014), including all 112 resident bird species in Inner Mongolia. Five thousand pseudoposterior distributions were sampled, and the maximum clade credibility tree was constructed using mean node heights by the software TreeAnnonator v1.8.2 of the BEAST package (Drummond & Rambaut, 2007; Ricklefs & Jønsson, 2014; Si et al., 2017). We used the resulting consensus phylogeny for all subsequent phylogenetic analyses. Phylogenetic relatedness of bird assemblages was represented by the Net Relatedness Index (NRI) (Webb, Ackerly, McPeek, & Donoghue, 2002). NRI is computed as

$$\text{NRI}=-1\times \frac{{\text{MPD}}_{\mathrm{obs}}-{\text{meanMPD}}_{\mathrm{rnd}}}{{\text{sdMPD}}_{\mathrm{rnd}}}$$

where MPD_obs_ is the observed mean phylogenetic distance (MPD) of birds in a county, meanMPD_rnd_ is the mean MPD of the null models (shuffle distance matrix labels 999 times), and sdMPD_rnd_ is the standard deviation of MPD of the null models. Positive NRI means birds in a county are more closed related than expected (clustered), while negative NRI means birds in a county are more distantly related than expected (overdispersed) (Webb et al., 2002). According to the phylogenetic niche conservatism hypothesis, a clustered phylogenetic structure indicates a dominant role of environmental filtering, and an overdispersed phylogenetic structure is driven by competition or facilitation (Webb et al., 2002).

### 2.4 Statistical analyses

Bird species richness, plant species richness, and county area were log transformed to obtain normal distributed residuals. All variables were standardized (mean = 0 and standard deviation = 1) to make the regression coefficients comparable. Relationships between bird species richness and each associated variable were then estimated by ordinary least squares (OLS) models. To account for spatial autocorrelation of residuals, simultaneous autoregressive (SAR) models were also used for the single‐variable analyses. Because Random Forest models could effectively capture interactions (e.g., in this study correlation between MAP and change in MAP is 0.76, and correlation between MAT and change in MAT is −0.70) and nonlinear relationships, and do not require the data to follow strict assumptions, for example, homoscedasticity and normality in errors (Breiman, 2001), they were implemented for the multiple‐variable analyses, aiming to find the combination of variables most associated with bird species richness. For each combination, the Random Forest models were run 1,000 times on random splits of the data (50% training data and 50% evaluation data) and averaging the Pearson correlation between the predicted and the observed values (species richness of birds). To check which variables always occurred in the best combinations, the six combinations with highest Pearson correlations were chosen. SAR models were also conducted for the six combinations because of spatial autocorrelation of residuals. AIC_w_ and *r* ^2^ of SAR models were listed for the six combinations. AIC_w_ is interpreted as the probability of each model being the best model and indicates the relative merit of the competing models (Wagenmakers & Farrell, 2004). OLS, SAR, and Random Forest models, as well as data transformations, were performed in R 3.3.0 (R Core Team, 2016) using vegan (Oksanen et al., 2015), spdep (Bivand et al., 2015), and randomForest (Liaw & Wiener, 2002) R packages.

## 3 RESULTS

**Figure 1:**
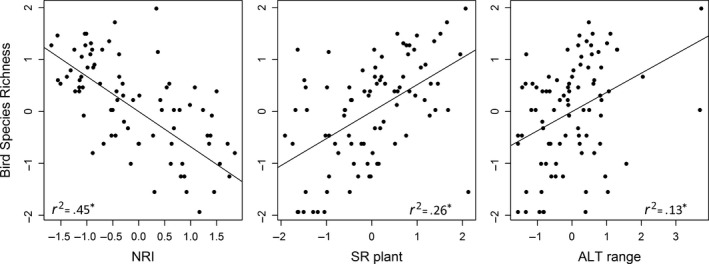
Relationships between bird species richness (log transformed) and the three most associated variables: phylogenetic relatedness of bird assemblages (Net Relatedness Index, NRI), plant species richness (SR plant, log transformed), and altitudinal range (ALT range). Single‐variable OLS linear fits (standardized) are shown, and their *r* ^2^ is given. All statistically significant *p*‐values were less than .01 and are indicated as *

The single‐variable ordinary least squares models and simultaneous autoregressive models showed similar patterns about the relationships between bird species richness and the associated variables (Table 1). The three variables significantly correlated with bird species richness were phylogenetic relatedness of bird assemblages (negatively, *r* ^2^ = .45), plant species richness (positively, *r* ^2^ = .26), and altitudinal range (positively, *r* ^2^ = .13), indicating that there were more birds with more overdispersed phylogenetic structure, more plant species, and larger altitudinal range (Figure 1, Table 1). Other variables, that is, contemporary climate and contemporary‐Last Glacial Maximum (LGM) change in climate, were not significantly correlated with bird species richness (Table 1).

Random Forest analyses showed that the six combinations of variables most associated with bird species richness consistently included phylogenetic relatedness of bird assemblages, plant species richness, and contemporary‐LGM change in MAT, indicating that although contemporary‐LGM change in MAT was not significantly correlated with bird species richness, it may still play a supplementary role in predicting distribution of bird species richness (Table 2).

**Table 2:** The six combinations of variables most associated with bird species richness, ranked by the correlations between observed and predicted species richness, from the Random Forest models (Cor_RF_). Each column is a different variable (NRI, phylogenetic relatedness of bird assemblages; Change_MAT_, contemporary‐Last Glacial Maximum change in temperature; SR_plant_, species richness of plant; MAP, mean annual precipitation; ALT_range_, altitudinal range; MAT, mean annual temperature; Change_MAP_, contemporary‐Last Glacial Maximum change in precipitation). White cell indicates that the variable was not included in the particular combination (each row). AIC weights (AIC_w_) and adjusted *r* ^2^ from simultaneous autoregressive (SAR) models of each combination of variables were also listed

NRI	Change_MAT_	SR_plant_	ALT_range_	MAP	MAT	Change_MAP_	Cor_RF_	AIC_w_SAR_	*r* ^2^_SAR_
							0.620	0.100	.544
							0.614	0.049	.547
							0.610	0.074	.551
							0.605	0.049	.557
							0.604	0.045	.546
							0.604	0.020	.548

## 4 DISCUSSION

It has been reported that the geographic distributions of bird species richness are shaped by divergent factors, including local, regional, and continental variables (Heikkinen et al., 2007; Qu et al., 2015; Rahbek & Graves, 2001). However, few studies have simultaneously tested the relative roles of a comprehensive set of the potential factors, especially in an arid and semiarid region with diverse vegetation types and species richness. Our results indicated an important role of local ecological factors, likely linked to vegetation diversity or productivity, environmental heterogeneity, and possibly, competition, in addition to the broad‐scale past and present climate factors.

### 4.1 Net relatedness index and bird species richness

A study of tropical humming birds communities found overdispersed phylogenetic structure (more distantly related than expected) in wet lowlands and clustered phylogenetic structure (more closely related than expected) at high altitude, and interpreted this as evidence of the strong influence of competition and environmental filtering in these two habitats (Graham, Parra, Rahbek, & McGuire, 2009). In this study, our analyses showed an increasing overdispersion of phylogenetic structure with higher species richness, potentially indicating a role of competition in shaping the build‐up of species‐rich bird assemblages and a role of environmental filtering on phylogenetically conserved traits in species‐poor assemblages (Figure 1). Being a fundamental process in ecology, competition is an important driver of both local community assembly and macro‐ecological scales species distribution (Feng et al., 2016; Fine, 2015).

### 4.2 Plant species richness and bird species richness

Positive correlations between plant species richness and bird species richness at regional scales have been widely reported, for example, in western Canada (Zhang et al., 2013), sub‐Saharan Africa (Kissling, Rahbek, & Böhning‐Gaese, 2007), and China (Qian & Kissling, 2010). Consistent with these studies, we also found positive correlations between bird species richness and plant species richness (Figure 1, Table 1). It is possible that higher plant species richness could provide more diverse habitats and food supplies for birds, thereby supporting more bird species (Zhang et al., 2013). It is also possible that there could be other factors (biotic and abiotic) affecting the diversity of birds and plants in similar ways (Kissling et al., 2007).

### 4.3 Environmental heterogeneity and bird species richness

Environmental heterogeneity is also an important driver of geographic distribution of species richness for different taxa, biomes, and spatial scales (Stein et al., 2014). The increase in environmental heterogeneity could provide more niches, refuges, and opportunities for speciation (Stein et al., 2014). Being an important and easily quantified proxy of environmental heterogeneity, altitudinal range has been widely related to bird species richness at macro‐ecological scales (Davies et al., 2007; Jetz & Rahbek, 2002; Jiménez‐Alfaro, Chytrý, Mucina, Grace, & Rejmanek, 2016). Consistent with these studies, we also found that bird species richness in Inner Mongolia increases with altitudinal range (Figure 1). Two counties with high bird species richness in our study, which are Alxa Left Banner (59 species) and Hexigten‐Banner (39 species), have high altitudinal ranges (2,243 m and 1,139 m). Most of these species (75% and 72%) prefer to live in forests. These two counties also have high species richness of plants (768 and 597 species), again emphasizing the importance of habitat heterogeneity in shaping the distribution patterns of bird species richness.

### 4.4 Climate and bird species richness

The refuge hypothesis assumes that stable glacial–interglacial climate change may both facilitate speciation and restrict extinction, thus regions with stable climate may have more species due to the accumulation of both relict and new species (Fjeldså & Lovett, 1997). Correlations between glacial climate fluctuation and bird species richness have been tested in Africa, Australia, and the New World (Fjeldså & Lovett, 1997; Hawkins, Diniz‐Filho, Jaramillo, & Soeller, 2006; Hawkins, Diniz‐Filho, & Soeller, 2005). Our analyses showed no significant correlations between bird species richness and historical climate variables (Table 1). However, the contemporary‐Last Glacial Maximum change in temperature occurred in all six of the most informative combinations of variables for bird species richness (Table 2), indicating that the glacial–interglacial climate change may have left a potential legacy in this region and may play a supplementary role in shaping the distribution of bird species richness.

Numerous previous studies have also examined the correlations between contemporary climate and bird species richness to test the energy availability hypothesis and the water–energy dynamics hypothesis (Jetz & Rahbek, 2002; Rahbek & Graves, 2001). However, our analyses did not show significant correlations between contemporary climate variables and bird species richness. Contemporary climate may affect the distribution of bird species richness through their effects on the distribution of vegetation and plant diversity in Inner Mongolia (Wu et al., 2015).

## 5 CONCLUSIONS

Through investigating the patterns and underlying drivers of the geographic distribution of bird species richness in Inner Mongolia, we found that local ecological factors, for example, environmental heterogeneity, habitat filtering, and biotic interactions, were associated with bird species richness, while regional and historical factors, that is, contemporary and historical climate, were not. Our findings highlight the importance of these local ecological factors in biodiversity maintenance.

## CONFLICT OF INTEREST

None declared.

## AUTHOR CONTRIBUTIONS

CL collected the data and drafted the article. GF designed the study, analyzed the data, drafted, and revised the article. XS and LM provided data and revised the article. GY, J.‐C.S., and JY revised the article.
